# Spontaneous confocal Raman microscopy--a tool to study the uptake of nanoparticles and carbon nanotubes into cells

**DOI:** 10.1186/1556-276X-6-429

**Published:** 2011-06-16

**Authors:** Gabriela Romero, Elena Rojas, Irina Estrela-Lopis, Edwin Donath, Sergio Enrique Moya

**Affiliations:** 1 CIC biomaGUNE, Paseo Miramón 182 C, 20009 San Sebastian, Spain; 2 Institute of Biophysics and Medical Physics, University of Leipzig Härtelstraße 16-18, D-04107 Leipzig, Germany

## Abstract

Confocal Raman microscopy as a label-free technique was applied to study the uptake and internalization of poly(lactide-*co*-glycolide) (PLGA) nanoparticles (NPs) and carbon nanotubes (CNTs) into hepatocarcinoma human HepG2 cells. Spontaneous confocal Raman spectra was recorded from the cells exposed to oxidized CNTs and to PLGA NPs. The Raman spectra showed bands arising from the cellular environment: lipids, proteins, nucleic acids, as well as bands characteristic for either PLGA NPs or CNTs. The simultaneous generation of Raman bands from the cell and nanomaterials from the same spot proves internalization, and also indicates the cellular region, where the nanomaterial is located. For PLGA NPs, it was found that they preferentially co-localized with lipid bodies, while the oxidized CNTs are located in the cytoplasm.

## Introduction

The use of nanomaterials and nanoparticles (NPs) in medicine as drug delivery vectors, sensors or contrast agents is among the most promising areas in nanotechnology research. For the application of nanotechnology in medicine, '*in vitro*' work is of paramount importance, especially regarding the assessment of possible toxicological consequences of the nanomaterials/NPs. It is a key issue to study the effects of 'nano' in the cellular machinery and to understand how the nanomaterials are processed in the cell, and their distribution and fate, after being taken up by the cells. Confocal laser scanning microscopy (CLSM) is often applied for uptake studies, but its application for NPs is not always easy, since the size of the NPs falls well below optical resolution. Also, a main drawback of CLSM is that, for most of the cases, both the NPs and cellular compartements must be fluorescently labelled, and this is not always an easy task. Besides that, labelling of NPs may in some cases require complex chemical routes including, for example, silanization, assembly of polymers, etc. As a result, the labelling can induce significant changes in the structure and properties of NPs, which may affect uptake and toxicity. Transmission electron microscopy (TEM) can be used to study the uptake and localization of NPs and nanomaterials, avoiding their labelling. The drawback of TEM for this application is that it requires complex and time-demanding preparations that also may affect the localization of the nanomaterials within the cell. Other label-free techniques for the study of the localization of nanostructures within cells are spontaneous Raman microscopy and coherent anti-stokes Raman (CARS) microscopy. In CARS, a single Raman band coming from the nanomaterial is scanned throughout the cell. A mapping of the cell is obtained showing the intensity distribution of the chosen Raman band [1]. Drawbacks of CARS are that only selected bands can be mapped, and furthermore, spectral overlapping may cause problems.

Confocal Raman microscopy (CRM) combines spontaneous Raman emission with confocal detection. We will show here that CRM can be used to study the localization of nanomaterials in the cells, taking advantage of the fact that in every spot the whole Raman spectrum is recorded. The latter thus contains bands coming from the nanomaterials and from representative cell molecules: proteins, DNA and lipids, which allow to identify the region of the cell [2,3], where the nanostructures are located. This article is among the first [4] to explore the use of the spontaneous Raman emission for the detection of nanomaterials inside cells and to assess the intracellular region from the spectra, where the nanomaterial is located. Previous study with CRM and cells has focused in the recognition of different cellular environment through their chemical fingerprints and the evaluation of changes in metabolism [5,6]. Poly(lactide-*co*-glycolide) (PLGA) NPs and carbon nanotubes (CNTs) have been chosen as two representative and remarkably different systems that can be studied with CRM. To our knowledge, this is the first article where CRM is used for CNT detection at cellular level.

## Materials and methods

PLGA (d,l-lactide 85,: glycolide 15, inherent viscosity within 0.55-0.,75 dL/g) was purchased from LACTEL^®^. Branched PEI, M_w _25 kDa, BSA, PIERCE BCATM Protein Assay Kit, Dulbecco's Modified Eagle's Medium (DMEM), fetal bovine serum and penicillin-streptomycin were purchased from Sigma-Aldrich. All chemicals were used as received. Hepatocarcinoma human cell line (HepG2) was obtained from American Type Culture Collection (ATCC-HB-8065).

PLGA NPs were prepared by the O/W emulsion-solvent evaporation method [4]. Size and shape of the PLGA NPs were characterized by TEM (JEOL JEM 2100F, Japan) [7]. Multiwalled CNTs were purchased from Proforma (USA). Oxidation of CNTs was achieved as described in the literature by Zhang et al. [8].

## Raman microscopy

Micro-Raman analyses were performed using a Renishaw inVia Raman Microscope. Measurements were performed using a 532-nm laser excitation wavelength with a grating of 1800 mm^-1^. Most measurements were conducted using a ×40 water immersion objective with a focal spot of approximately 1 μm in diameter. Spectra were recorded in the region 300-3600 cm^-1 ^, with a resolution of approximately 7 cm^-1^. The system was calibrated to the spectral line of crystalline silicon at 520.7 cm^-1^. At least 8-15 accumulation scans, at different spots in the various cell compartments, lipid bodies (LB), cytoplasm and nucleus, were used to reduce the spectral noise. All spectra had a correction for the PBS solution and glass cover slip baseline. After CNTs or NPs exposure and repeated washings with PBS, the Raman spectra were taken only from cells, where no visible CNT aggregates could be observed.

## Results

**Figure 1:**
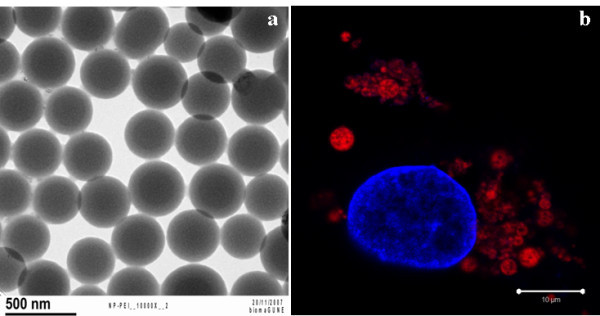
**Characterization and confocal fluorescence imaging of PLGA NPs in cells**. (a) TEM morphology of PLGA NPs stabilized with PEI and (b)CLSM images of HepG2 cells after being co-cultured with PLGA NPsstabilized with PEI.

In Figure 1a, a characteristic TEM image of the PLGA NPs is shown--the size of the NPs ranges from approximately 250-400 nm. PLGA NPs were labelled with Rd6G for visualization in HepG2 cells with CLSM. In Figure 1b, the confocal images show that PLGA NPs are associated with the cells. This follows from the red colour indicating the Rd6G-labelled NPs, distributed around the blue-stained nucleus. The single confocal image does not unambiguously prove the internalization of the PLGA NPs in the cells, which could also be associated to the cell membrane. However, a scan in the *z*-direction (*z*-scan) could show the intracellular presence of the NPs, especially, if the plasma membrane had been also stained [9].

**Figure 2:**
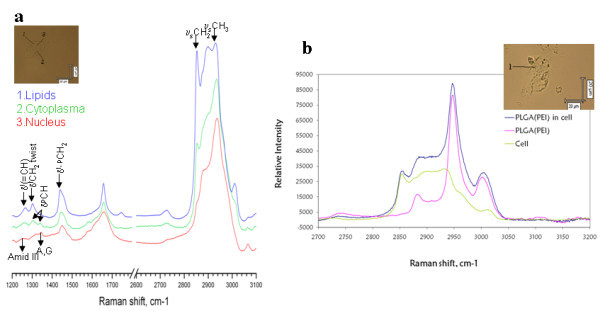
**Confocal Raman imaging of HepG2 cells before and after exposure to PLGA NPs**. (a) Raman spectrum recorded at different positions within a cell from the HepG2 line (ν indicates stretching and δ deformation vibration modes; l denote vibrations of lipids and p of protein). (b) Spot Raman spectra (dark blue) in cells exposed to PLGA NPs covered with PEI, pink and green lines denote the component spectra of PLGA NPs and of the cells. The insets correspond to the image of the cell under study

CRM experiments were performed in the same experimental conditions, but without labelling of the NPs, and are shown in Figure 2. In Figure 2a, representative Raman spectra, taken at different regions of the cell are shown. The nucleus, cytoplasm and LB can be identified by their chemical signature provided by the Raman spectrum. The symmetric stretch bands of CH_2 _(2850 cm^-1^) to CH_3 _(2935 cm^-1^) is much more intense in LB (blue line) than in the cytoplasm (green line) due to a lower density of CH_2 _groups in proteins compared with lipids. The nucleus region was identified by the smallest intensity ratio of CH_2 _to CH_3 _bands, as well as by bands assigned as vibration of DNA bases of adenine (A) and guanine (G) (red line). The spectra in Figure 2b correspond to Raman spectra taken from HepG2 cell, before and after incubation with PLGA NPs. A spectrum of the PLGA NPs taken in the dry state is also shown (pink curve). It can be seen that the Raman spectrum of the cells, after incubation with PLGA, represents a superposition of the PLGA particle spectrum (pink) and the spectrum of the control (green). Besides the bands typical from lipids at 2850 and 2900 cm^-1^, which can be attributed to the LB, the intense CH_2 _and CH_3 _vibrations of PLGA are clearly visible. Looking at different spots in the cell, at the same plane and at different positions regarding the *z*-direction, revealed that when bands characteristic for PLGA NPs were observed, the typical LB signature was also present.

**Figure 3:**
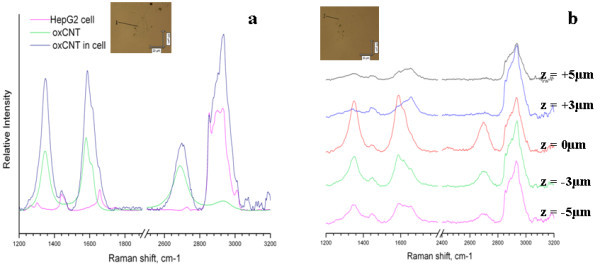
**Raman spectra of CNTs and Raman imaging of cells exposed to CNTs**. (a) Spectra of oxidized CNTs (green), free cell in the region of the LB (pink) and cell exposed to CNTs at the same region (blue). (b) Raman spectra taken in one spot at different planes at a HepG2 cell treated with oxidized CNTs. The insets correspond to the image of the cell under study.

Similar uptake experiments were performed with oxidized CNTs. The CNTs were oxidized to provide them with charges to ensure their stabilization in aqueous solution. In Figure 3a, we can observe the Raman spectrum of oxidized CNTs and HepG2 cells exposed to the CNTs. CNTs show characteristic bands at 1350 cm^-1 ^(D-band) and 1585 cm^-1 ^(G-band) [10,11]. The D-band is an indicator for disorder in the graphene sheet and is called ''disorder-induced" band. The G-band is a tangential mode originating from tangential oscillations of the carbon atoms in the CNTs. These bands can be clearly observed in the cellular spectra. Scans were performed at different planes within the cells as shown in Figure 3b. The plane denoted by 0 μm corresponds to the situation, where the signals of the D and G bands from the CNTs were the strongest. Then, spectra were recorded at higher and lower planes, respectively. In all cases, the CNTs spectral signature was parallelled by CH_3_-stretching modes, typical for proteins. The CH_2 _stretching, which is indicative for LB, can be barely seen. The intensity of the CNTs bands varied considerably over the different scan planes. From these findings, we draw the conclusion that the CNTs are not homogeneously distributed in the cytoplasm, nor they are closely associated with LB. Furthermore, the *z*-scanning provides an unambiguous proof of internalization of the NPs, since we move in distances of micrometers inside the cell, where the detection of CNTs attached to the cell membrane from the outside is very unlikely.

## Conclusions

Spontaneous CRM has been successfully applied to identify PLGA NPs and oxidized CNTs in single hepatocarcinoma cells, which had been co-cultivated with the NPs and CNTs. The data prove that CRM, being a label-free technique, is a valuable tool to study the uptake of nanomaterials into cells. For PLGA NPs, CRM confirms the observations from CLSM and proves internalization. The *z*-scanning of the cells with CNTs reveals that these are incorporated in the cytoplasm and are not co-associated with the LB.

## Abbreviations

CARS: coherent anti-stokes Raman; CLSM: confocal laser scanning microscopy; CNTs: carbon nanotubes; CRM: confocal Raman microscopy; LB: lipid bodies; NPs: nanoparticles; PLGA: poly(lactide-*co*-glycolide); TEM: transmission electron microscopy.

## Competing interests

The authors declare that they have no competing interests.

## Authors' contributions

GR performed the synthesis of the PLGA nanoparticles and the surface modification of the CNTs. She also conducted the Confocal Raman experiments. ER conducted the Confocal Fluorescence Microscopy experiments and did the cell culture work. IE supported GR with the Raman experiments and helped with the interpretation of the data. ED provided support in the design of the experiments and the interpretation of the Raman spectra. SEM conceived, designed and coordinated the study. All authors read and approved the final manuscript.
